# Membrane proteome of the thermoalkaliphile *Caldalkalibacillus thermarum* TA2.A1

**DOI:** 10.3389/fmicb.2023.1228266

**Published:** 2023-07-28

**Authors:** Samuel I. de Jong, Dimitry Y. Sorokin, Mark C. M. van Loosdrecht, Martin Pabst, Duncan G. G. McMillan

**Affiliations:** ^1^Department of Biotechnology, Delft University of Technology, Delft, Netherlands; ^2^Winogradsky Institute of Microbiology, Research Centre of Biotechnology, Russian Academy of Sciences, Moscow, Russia

**Keywords:** (Poly-) extremophile, thermophile, alkaliphile, membrane, proteomics, mass spectrometry, solubilisation

## Abstract

Proteomics has greatly advanced the understanding of the cellular biochemistry of microorganisms. The thermoalkaliphile *Caldalkalibacillus thermarum* TA2.A1 is an organism of interest for studies into how alkaliphiles adapt to their extreme lifestyles, as it can grow from pH 7.5 to pH 11. Within most classes of microbes, the membrane-bound electron transport chain (ETC) enables a great degree of adaptability and is a key part of metabolic adaptation. Knowing what membrane proteins are generally expressed is crucial as a benchmark for further studies. Unfortunately, membrane proteins are the category of proteins hardest to detect using conventional cellular proteomics protocols. In part, this is due to the hydrophobicity of membrane proteins as well as their general lower absolute abundance, which hinders detection. Here, we performed a combination of whole cell lysate proteomics and proteomics of membrane extracts solubilised with either SDS or FOS-choline-12 at various temperatures. The combined methods led to the detection of 158 membrane proteins containing at least a single transmembrane helix (TMH). Within this data set we revealed a full oxidative phosphorylation pathway as well as an alternative NADH dehydrogenase type II (Ndh-2) and a microaerophilic cytochrome oxidase *ba*_3_. We also observed *C. thermarum* TA2.A1 expressing transporters for ectoine and glycine betaine, compounds that are known osmolytes that may assist in maintaining a near neutral internal pH when the external pH is highly alkaline.

## Introduction

The biological membrane is where the cell meets its environment, forming the boundary between the cytoplasm and the outside world and also at the point where the cell senses environmental changes. They are composed of proteins, lipids, lipid-like molecules, and sugars. The way in which the cell senses these changes is through membrane-embedded and membrane-associated proteins (Boes et al., 2021). Proteins in the membrane have diverse functions, from the obvious bioenergetic processes, such as facilitating the active transport of useful solutes into the cell, to broader regulatory functions. The dual function is also common, such as for an enzyme found in the membrane-bound pathway for cellular respiration, an enzyme known as the F_1_F_o_ ATP synthase. On the one hand, its purpose is to harness both a proton or sodium gradient alongside membrane potential to regenerate ATP, a form of chemical cellular energy; on the other hand, it is also thought to regulate cytoplasmic pH (Krah et al., 2018).

The membranes of extremophiles are even more important as a physical barrier, as they have a much more protective role against the adverse environment outside the cell. For example, conditions including high concentrations or high temperatures, high salinity, extreme acidity, or alkalinity are common in extreme environments. Thermophiles adapt by producing more saturated lipids or by branching fatty acids, and sometimes lipids may have complex structures (Reizer et al., 1985; Russell and Fukunaga, 1990; Sinninghe Damsté et al., 2011), increasing their rigidity (Russell and Fukunaga, 1990). With alkaliphiles, the defining environmental pressure is a lack of environmental proton availability. This leads to a situation where there is a lower pH inside the cell than outside the cell – a so-called “inverted pH gradient.” In aerobic alkaliphiles, this is a mountainous challenge and a complete bioenergetics conundrum because cellular energy generation is coupled to this inverted proton gradient (Krulwich, 1995; Horikoshi, 2004; McMillan et al., 2007). When these two environmental pressures are combined in a given organism, the membrane is theoretically very sensitive to leakage, so it must prevent the loss of protons to the outside environment (Takami, 2000; Yumoto, 2002; Olsson et al., 2003; McMillan et al., 2007). Adaptations to minimize proton leakage are localized to the membrane (Koch, 1986; Krulwich, 1995). The architecture of the lipid bilayer is important for its protective properties, but for all other membrane processes, proteins are involved. Adaptations to cope with this environment are proposed to include a highly saturated and branched lipid composition (Siliakus et al., 2017), making solubilisation of such membranes to extract membrane proteins for downstream analysis a challenging process. This situation is a “nightmare scenario” in terms of a proteomics approach to understanding environmental adaptation at the cell membrane.

Proteomics of membrane proteins is challenging in general. The typical protocol for membrane protein detection and quantification relies on proteolytic digestion of soluble or solubilised proteins, followed by liquid-chromatography linked to mass-spectrometry (MS) detection. Subsequently, the processing of the data obtained is delineated using various database search tools. This yields a tremendous amount of information but invariably underrepresents the membrane proteins (Sievers, 2018). Most of the transmembrane proteins harbor a large number of hydrophobic TMHs. The hydrophobic nature of these proteins makes them insoluble in aqueous environments, a particular challenge in MS detection. Moreover, TMHs are protected from proteolytic digestion, a key requirement to generate peptide fragments for MS detection. With these proteins, only the short loops connecting such helices and soluble domains are accessible for proteolytic cleavage. To add to these challenges, membrane proteins suffer from low absolute expression levels because membrane “real estate” is severely limited (Fischer et al., 2006; Helbig et al., 2010). Solubilisation of membrane extracts is a potential approach for the extraction of integral membrane proteins, yet defining a universal protocol for that solubilisation is seemingly impossible (Schuck et al., 2003; Eshaghi et al., 2005). A key reason for the problems in building such a protocol is that every organism constructs its membrane to suit the conditions under which it grows, i.e., an organism will adapt the membrane composition to avoid compromising on membrane fluidity or polarity (Uda et al., 2001; Meador et al., 2014; Mendoza, 2014). In general, the literature on solubilising membranes for proteomics focuses on well-studied model organisms, such as *Escherichia coli* (Eshaghi et al., 2005). The field of membrane proteomics in extremophiles is poorly developed. For thermophiles, a few studies also focus on this subject. Two published works detail whole-cell studies in *Clostridium thermocellum* (Williams et al., 2007; Burton and Martin, 2012), and membrane solubilisation of the archaeon *Sulfolobus solfataricus* is summarized (Zaparty et al., 2009). For alkaliphiles, two studies have been reported. One investigates membrane solubilisation with urea in *Alkalihalobacillus marmarensis* (Altinisik Kaya et al., 2018), while the other focuses on a few other well-known yet ultimately ineffective detergents for *Alkalimonas amylolytica* (Wang et al., 2009). None of these studies rely on a comparative approach that assesses the contribution of each step to the protocol. Here, we attempted to address this shortcoming and advance the field of membrane proteomics for polyextremophiles.

In this study, we focus on a polyextremophile, the thermoalkaliphile *Caldalkalibacillus thermarum* TA2.A1. This microorganism is highly adapted to the harsh conditions that it lives in (pH 9.5 at 65°C) (Peddie et al., 1999) and has a high degree of variability in its ETC (de Jong et al., 2020). We reveal the membrane proteome by implementing different solubilisation techniques while considering the organism's physiological growth temperature. Specifically, we present an overview of the detected membrane proteome of *C. thermarum* TA2.A1, focusing particularly on its transporters and ETC. Our findings also resulted in a noteworthy increase in detectable proteins and peptides.

## Materials and methods

### Cultivation and harvesting of biomass

*Caldalkalibacillus thermarum* TA2.A1 was grown in 5-L flat-bottomed shake flasks at 65°C with a working volume of 1 L rich medium, the composition of which was described previously (McMillan et al., 2009). A smaller pre-inoculum was grown beforehand in a 500-ml round-bottomed shake flask with a 100-ml working volume. The larger shake flasks were inoculated at 1% from the pre-inoculum. The incubations lasted for ~18 h. Cells were harvested by centrifugation at 8,000 × *g* and were subsequently washed. The fraction appropriated to whole cell proteomics (±100 mg cell wet weight) was washed in ice-cold phosphate-buffered saline (PBS) and thereafter flash frozen with liquid nitrogen and stored at −80°C. The fraction used for membrane proteomics was washed in 50 mM Tris–HCl buffer, pH 8.0, with 10% (w/v) glycerol and 2 mM MgCl_2_, and thereafter also flash frozen and stored at −80°C.

### Membrane extraction and solubilisation

Membrane preparation was performed as described earlier (McMillan et al., 2007), with modifications. Briefly, samples were thawed gradually by placing them on top of a layer of ice. Extraction started with a lysis step in 50 mM Tris-HCl buffer, pH 8.0, with 10% (w/v) glycerol, 2 mM MgCl_2_, 1 mM phenylmethylsulphonyl fluoride, and 1 mM dithiothreitol. Lysis was initiated by the addition of 1.2 mg ml^−1^ lysozyme and 0.2 mg DNaseI. Lysed cells were subsequently passed through a cell disruptor twice at 20,000 psi. As a trial, the number of passages through the cell disruptor was decreased to a single passage, but that yielded no discernible difference (Supplementary Figure 1). The resulting liquid fraction, discarding the denatured fraction, was centrifuged at 8,000 × *g* for 10 min. The resulting supernatant was ultracentrifuged at 180,000 × *g* for 45 min. The supernatant was discarded, and the pellet was resuspended in ~2 ml of 50 mM Tris-HCl buffer, pH 8.0, with 10% (w/v) glycerol and 2 mM MgCl_2_. When required, extracted membranes were flash frozen and stored at −80°C. *C. thermarum* TA2.A1 membranes were solubilised in 200 μM ammonium-bicarbonate (ABC) buffer with either 2% (w/v) n-tetradecylphosphocholine (FOS-choline-12, Anatrace) or 0.01% (w/v) sodium dodecyl sulfate (SDS) for 30 min at 50°C, 65°C, or 80°C, while gently shaking at 300 rpm. Extracted membrane samples were diluted to a final concentration of 4 mg ml^−1^ protein. After solubilisation, samples were frozen at −20°C until further preparation.

### Whole-cell lysate preparation

The following protocols were based on extensive work done before (den Ridder et al., 2022, 2023) to optimize the protocols. For whole cell proteomics, the pellet stored after washing with PBS was thawed, and 29 mg of the sample was used and transferred to a LoBind Eppendorf tube. The pellet was dissolved in 0.175 ml of 1 M triethylammonium bicarbonate buffer, and 0.15 g of glass beads (105–212 μm, Sigma Aldrich) were added. The sample was subjected to a vortexing regime that was repeated thrice; the sample was vortexed at maximum speed for 90 s and then placed on ice for 30 s. Afterward, the sample was incubated at 80°C for 3 min at 1,000 rpm and subsequently ultrasonicated for 10 min. The sonicated sample was centrifuged, and the resulting supernatant (250 μl) was carefully pipetted into a new LoBind Eppendorf tube. The proteins were then precipitated by adding 62.5 μl of 6.1 N tricholoacetic acid by vortexing and subsequent incubation at 4°C for 30 min. After centrifugation, the resulting pellet was washed with 200 μl of ice-cold acetone and then centrifuged again. The washed pellet was redissolved in 50 μl of 6 M urea. The sample was then subjected to the proteolytic digestion protocol as described below.

### Proteolytic digestion

After thawing, in the case of the membrane extract, samples were transferred to a LoBind Eppendorf tube, vortexed, and an additional 50 μl of 200 mM ABC buffer was added. A total of 100 μl (≈100 μg protein) of that mix was used for reduction and alkylation. For the whole cell sample, proteolytic digestion occurred straight after lysate preparation. In this case, the preceding protocol yielded 30 μg of protein. For the following part, two volumes are given for every step; the lower volume corresponds to the whole cell sample and the higher volume to the membrane extracts. The reduction was performed by incubation with 8.5 or 30 μl of 10 mM dithiothreitol at 37°C for 60 min at 300 rpm. Alkylation was subsequently performed by adding 8.5 or 30 μl of 20 mM iodoacetamide and incubating at 20°C for 30 min in the dark. Samples were then further diluted with 285 or 400 μl ABC, and 5 or 20 μl of 0.1 μg μl^−1^ trypsin was added for digestion. Digestion occurred for ~18 h at 37°C and 300 rpm. After digestion, all samples were treated equally again. After digestion, peptides were extracted by solid-phase extraction in an Oasis HLB 96-well μElution Plate (Waters) according to manufacturer specifications. Peptides were eluted in two phases: (1) with 200 μl of 2% formic acid in 80% methanol and (2) with 200 μl of 1 mM ABC in 80% methanol. The peptides were then collected in an Integrated SpeedVac^TM^ System (Thermo Scientific) at 50°C for 2 h. Samples were stored at −20°C until analysis.

### Shotgun proteomics and raw data processing

Shotgun proteomic analysis was performed as described earlier (Kleikamp et al., 2021; Pabst et al., 2021). Speed vac dried samples were dissolved in 20 μl of 3% acetonitrile plus 0.1% trifluoroacetic acid. Protein concentrations were estimated by NanoDrop ND-1000 spectrophotometry (Thermo Scientific). An aliquot corresponding to ~0.5 μg of protein was analyzed using a one-dimensional shotgun proteomics approach using a nano-liquid chromatography system consisting of an EASY nano-LC 1200 (Acclaim PepMap RSLC RP C18 separation column, 50 × 150 mm, 2 μm, Cat. No. 164568) and a QE plus Orbitrap mass spectrometer (Thermo Fisher Scientific, Germany). The flow rate was maintained at 350 nl/min over a linear gradient from 5 to 25% solvent B over 88 min, then from 25 to 55% over 60 min, followed by back equilibration to starting conditions. Data were acquired from 5 to 240 min. Solvent A was H_2_O containing 0.1% formic acid, and solvent B consisted of 80% ACN in H_2_O and 0.1% FA. The Orbitrap was operated in data-dependent acquisition (DDA) mode, acquiring peptide signals from 385 to 1,250 m/z at 70 K resolution in full MS mode with a maximum ion injection time (IT) of 75 ms and an automatic gain control (AGC) target of 3E6. The top 10 precursors were selected for MS/MS analysis and subjected to fragmentation using higher-energy collisional dissociation (HCD). MS/MS scans were acquired at 17.5 K resolution with an AGC target of 2E5 and an IT of 75 ms, a 2 m/z isolation width, and normalized collision energy (NCE) of 28. Data were analyzed against the proteome database from *C. thermarum* [GenBank: CP082237.1, strain *Caldalkalibacillus thermarum* TA2.A1 complete genome, Tax ID: 559292 (de Jong et al., 2020)] using PEAKS Studio X (Bioinformatics Solutions Inc., Waterloo, Canada), allowing for 20 ppm parent ion and 0.02 m/z fragment ion mass error, three missed cleavages, carbamidomethylation and methionine oxidation, and N/Q deamidation as variable modifications. Peptide spectrum matches were filtered against 1% false discovery rates (FDR), and identifications with ≥2 unique peptides were considered significant. Results from PEAKS were exported to “proteins.csv,” listing the identified proteins.

Proteins from database searching files (Supplementary Table 1) were further processed using Python (version 3.9). Files were converted to “Pandas” dataframes and counted based on the following two criteria: “Area” > 0 and “Unique Peptides” ≥ 2. Venn diagrams were then visualized using the “Matplotlib” functions “*venn2_unweighted*” or “*venn3_unweighted*.” For the Venn diagrams concerning only membrane proteins, an additional criterion was added (TMH's ≥ 1). For the heat map, the raw absolute area was used directly, after filtering using Supplementary Table 2. The heat maps were visualized using the function ‘*vizus.gene_exp_hmap*' from the package ‘Bioinfokit'.

## Results and discussion

### Proteomics protocol optimisation for detection of *C. thermarum* TA2.A1 membrane proteins

**Figure 1:**
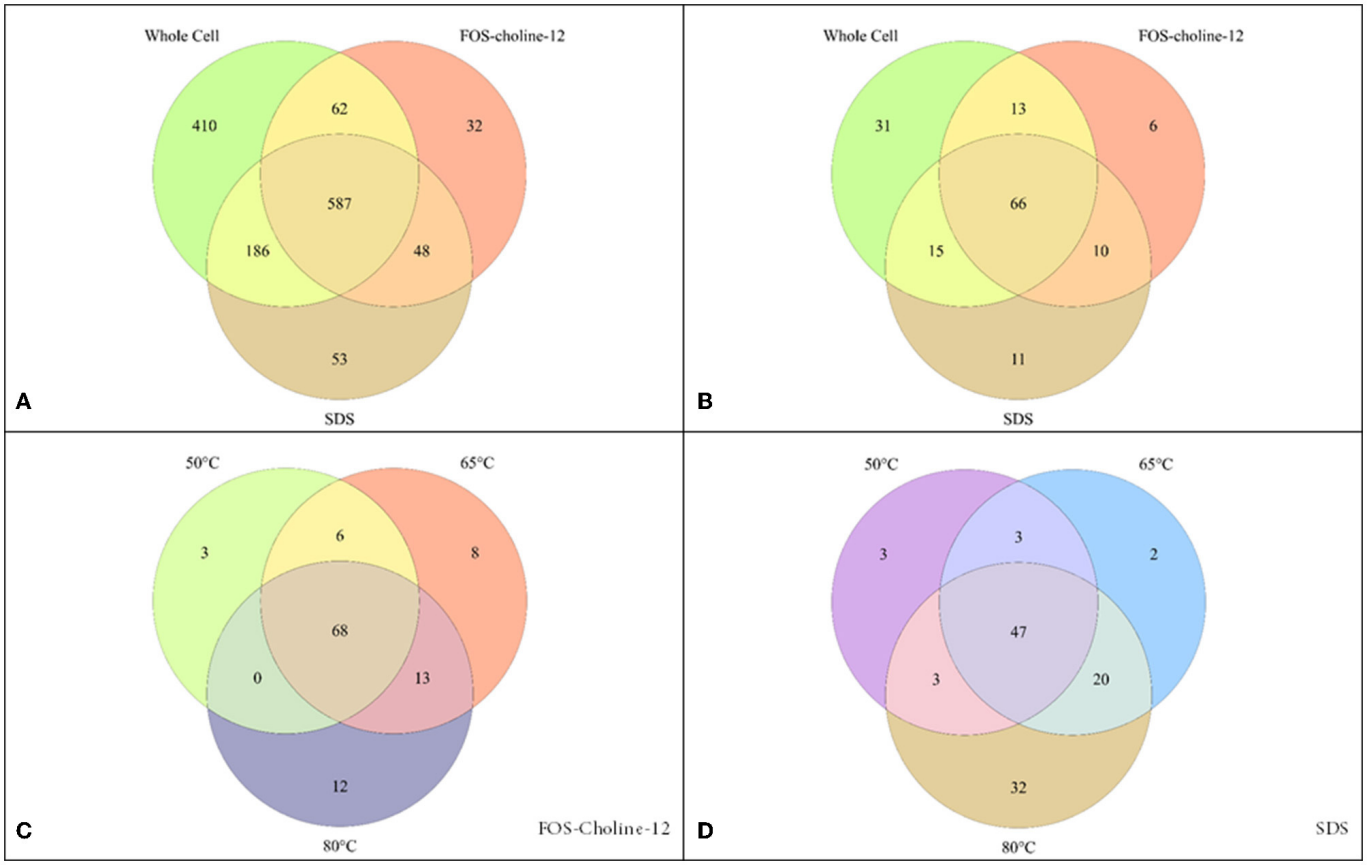
Detection of proteins by proteomics methods with different solubilisation methods. **(A)** covers the entire potential proteome, while **(B–D)** zoom in on solely the membrane proteins. **(A, B)** compare the detection using whole cell proteomics vs. that with solubilisation with FOS-choline-12 at 65°C and SDS at 80°C. **(C, D)** show the effect of solubilisation at different temperatures (50°C, 65°C, and 80°C) using either FOS-choline-12 **(C)** or SDS **(D)**.

We first performed a conventional whole-cell lysate shotgun proteomics experiment to detect the soluble protein fraction. The full genome of *C. thermarum* TA2.A1 (de Jong et al., 2020) contains 3,085 genes (NCBI; accession: CP082237), of which 1,244 (Figure 1A) were detected in this experiment (~40% of the genome). DTU's TMHMM tool was used to distinguish which of these proteins were likely integral membrane proteins, using a single TMH as the cut-off (Krogh et al., 2001). The *C. thermarum* TA2.A1 genome contains 754 proteins out of the total 3,085 that contain a single predicted TMH. It should be noted that proteins associated with the membrane (peripheral membrane proteins) but lacking a TMH are not “soluble proteins” and are also included in our analysis. Peripheral membrane proteins are membrane-associated *via* hydrophobic patches, lipid anchors, or lateral helices (Boes et al., 2021). However, these proteins cannot be identified from genetic information or be unequivocally identified or in a whole-cell proteome analysis without supporting biochemical data. Our analysis confirmed that only 124 of the 1,244 proteins detected contain a TMH. Considering that roughly a quarter of all proteins in the complete genome will be either integral or peripheral membrane proteins (Wallin and Von Heijne, 1998; Sawada et al., 2007), this reinforces the notion that the membrane proteome is generally underrepresented in conventional whole cell lysate proteomics experiments. Nevertheless, the whole-cell proteomics approach yields a membrane proteome with a considerable proportion of membrane proteins. For instance, the majority of the aerobic ETC proteins were detected. The integral membrane protein NADH dehydrogenase type I (Ndh-1) was detectable, as was the peripheral membrane protein NADH dehydrogenase type II (Ndh-2) (Godoy-Hernandez et al., 2019; Godoy-Hernandez and McMillan, 2021; Juergens et al., 2021). Other integral membrane proteins, such as succinate dehydrogenase (Sdh) and the cytochrome *b*_6_*c*_1_ complex (a hybrid complex III), were also detectable, as well as the cytochrome oxidase *aa*_3_ complex. The cytochrome oxidase *aa*_3_ complex is expected to be the primary terminal oxidase under fully aerobic conditions. However, we also detected a single subunit pertaining to the microaerophilic cytochrome oxidase *ba*_3_ complex. Surprisingly, the subunit detected contains 13 TMHs, while the soluble subunits were undetectable. However, caution should be taken with such a result because to confidently state that *C. thermarum* TA2.A1 expressed two terminal oxidases under the growth conditions we imposed, detecting the additional subunits provides greater clarity toward the notion of functionality. Finally, the ATP synthase was also detected, revealing that we can get very close to detecting a fully functional *C. thermarum* TA2.A1 pathway for oxidative phosphorylation from whole cell proteomics.

To better evaluate the possible expression of the cytochrome oxidase *ba*_3_, as well as many more functional proteins, increased detection is paramount. Unfortunately, membrane proteins are lesser in number in a cell, so a whole-cell proteome analysis including soluble proteins dwarfs the lower signals of membrane proteins (Tan et al., 2008). Isolating the membrane provides an initial step toward enrichment. Second, detergent solubilisation can singularly isolate proteins and is an easily accessible method to purify proteins for this reason (McMillan et al., 2007; Pullara et al., 2013; Mathieu et al., 2019). Proteins are in a crowded space in the biological membrane, and this crowding can prevent access by the proteases necessary for proteomics. Finally, as previously mentioned, for peripheral membrane proteins lacking biochemical description, we cannot rely on genetic information and cannot be unequivocally identified in whole-cell proteome analysis (Boes et al., 2021).

**Figure 2:**
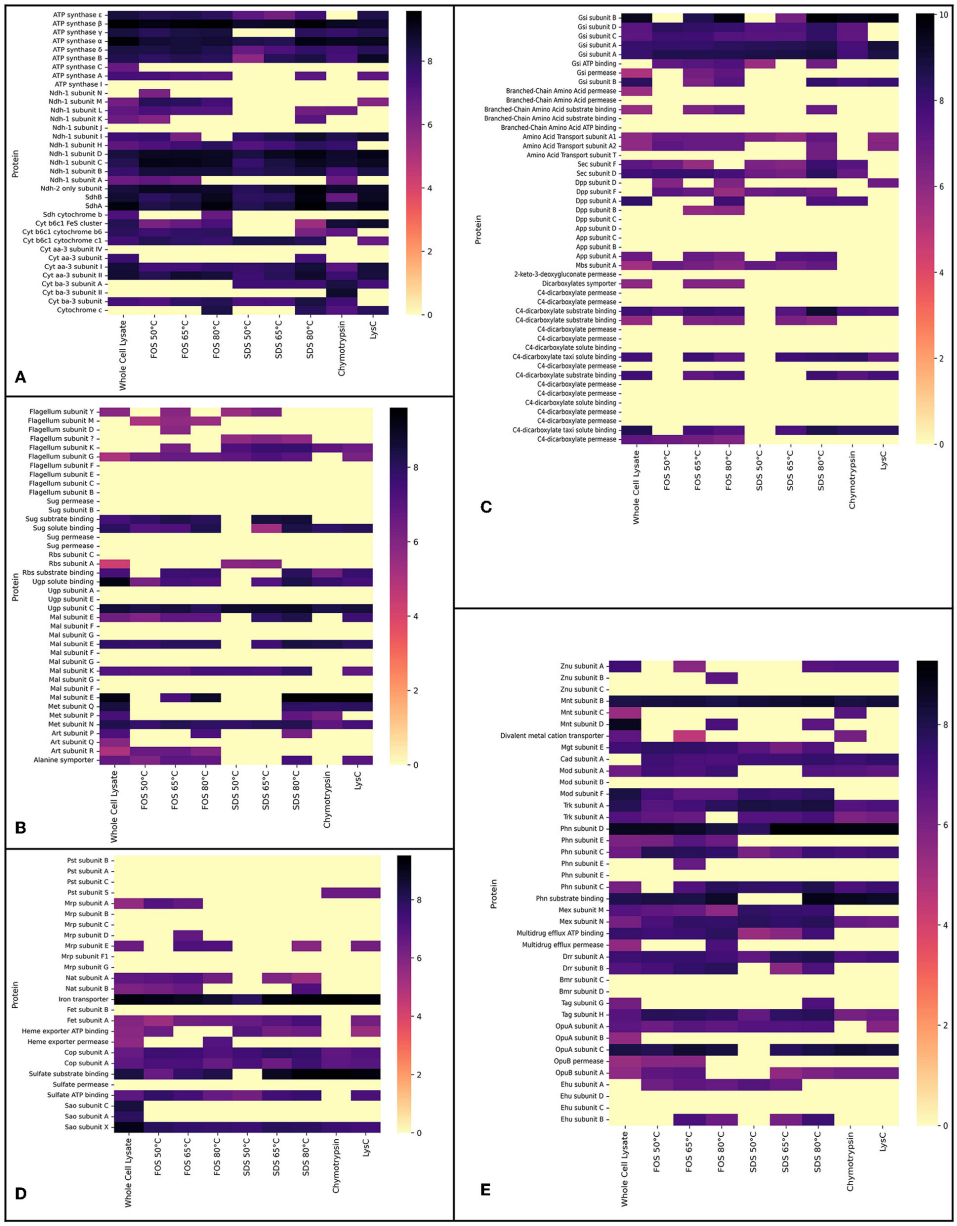
Proteomics data showing the logarithm (base 10) of absolute abundances detected in the whole cell lysate experiment and the experiments in which membrane extracts were solubilised with either FOS-choline-12 or SDS. For FOS-choline-12 and SDS, the temperature of solubilisation is given. Experiments with the alternative digestion enzymes, Chymotrypsin and LysC, are accrued together. **(A)** Heat map shows proteins of the ETC. **(B)** Heat map shows proteins of the flagellum and transporters of sugars amino acids. **(C)** Heat map shows transporters of primarily secondary metabolites; **(A–C)** together resemble heat maps corresponding to Figure 3. **(D, E)** Heat maps selectively show transporters of metals and other inorganic cations and anions, as in Figure 4. Heat maps show all subunits pertaining to a certain protein complex if at least a single subunit was detected in one of the conditions. Note that proteins absent here (but shown in both Figures 3, 4) were not detected by any method.

**Figure 3:**
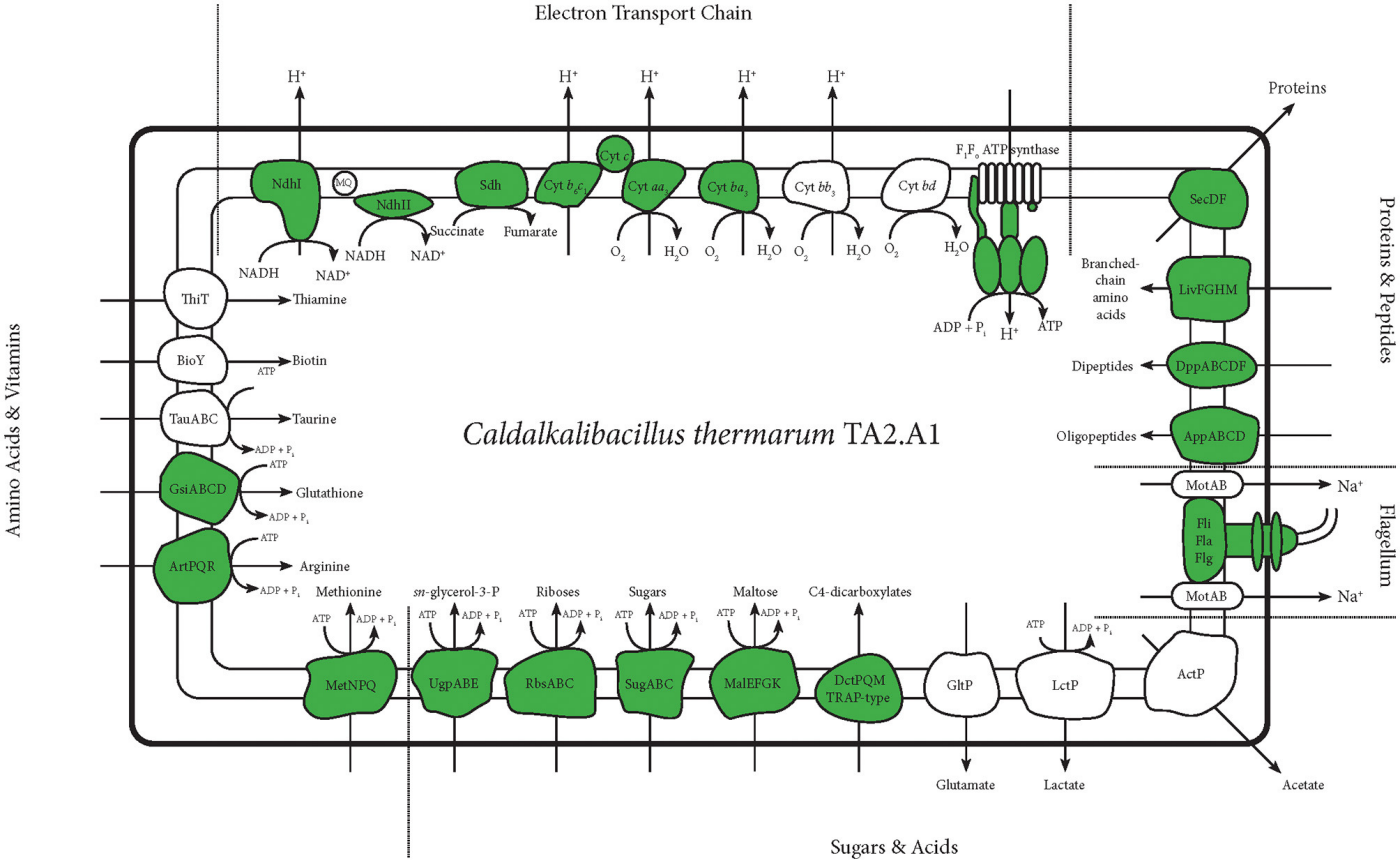
An overview of membrane proteins detected with any of the abovementioned protocols. This figure selectively shows all membrane proteins concerning the ETC and transporters concerning central carbon metabolism and anabolism. It also shows the flagellum. Detected proteins are depicted in green. For the ATP synthase and the flagellum, this figure is more detailed, and only the subunits detected are depicted in green.

To obtain better coverage of the membrane proteome, a modified membrane extraction protocol was followed. While extracting the membrane fraction from the rest of the cell constituents added a very modest increase in protein hits, adding a membrane solubilisation step significantly increased the detection of membrane proteins (Figures 1A, B). The breadth of solubilisation methods available in the literature (Schuck et al., 2003; Eshaghi et al., 2005; Williams et al., 2007; Wang et al., 2009; Zaparty et al., 2009; Burton and Martin, 2012; Altinisik Kaya et al., 2018) leaves a plethora of options available, but it is well noted that there is no common optimal method. Given our knowledge of membrane proteins, we opted for the use of detergents. Most detergents are not compatible with mass spectrometry, thus limiting options. Fortunately, two extremely effective detergents for membrane protein solubilisation were feasible [FOS-choline-12 and sodium dodecyl sulfate (SDS)]. Additionally, the effect of the temperature (50°C, 65°C, and 80°C) used for solubilisation was assessed. For solubilisation with FOS-choline-12, using 65°C or 80°C yielded optimal results, while for SDS, 50°C was clearly optimal (Figures 1C, D). Additionally, cross-comparison between the two detergents showed that each detergent appears to solubilise the membrane protein fraction selectively (Figure 1B). Finally, we tested whether changing the digestion enzyme made a difference in protein detection. An *in silico* digestion (Supplementary Figure 2) showed potential improvement in the optimal peptide length range using both LysC and chymotrypsin as digestion enzymes. Contrary to expectations, neither increased the detection of membrane proteins significantly, having only a modest effect (Figures 2, 3). Combining all proteomics experiments allowed for the construction of an overview of all detected proteins containing a TMH, as well as zooming in on the ETC and known transporters.

### An overview of the detected *C. thermarum* TA2.A1 membrane proteome

The main goal of our study was to identify as many membrane proteins as possible through the optimisation of membrane proteomics. In total, 158 membrane proteins were detected using the standard whole cell proteome method; although a full aerobic ETC was detected, not all subunits of each complex were found. Solubilisation enabled the detection of the other two remaining subunits pertaining to the microaerophilic cytochrome oxidase *ba*_3_ (Figure 2A). The aerobic and most efficient ETC *C. thermarum* TA2.A1 consists of Ndh-1, Sdh, cytochrome complex *b*_6_*c*_1_, cytochrome *c*, and cytochrome oxidase *aa*_3_. The expression of the F_1_F_o_ ATP synthase completes an aerobic oxidative phosphorylation pathway. All these complexes were detected (Figure 3).

Besides the expected aerobic ETC, somewhat surprisingly, ETC components that have been proposed to be more tuned toward a microaerophilic lifestyle, Ndh-2 and cytochrome oxidase *ba*_3_, were expressed under aerobic conditions. The *ba*_3_ complex proteins were among the few extra proteins detected after digestion with chymotrypsin or LysC instead of the standard trypsin digestion. The detection of Ndh-2 is in line with earlier research on this enzyme in *C. thermarum* TA2.A1, where it has been studied as a potential analog for drug targets (Heikal et al., 2014; Godoy-Hernandez et al., 2019; Godoy-Hernandez and McMillan, 2021). Finding the presence of cytochrome oxidase *ba*_3_ was more surprising, as it is only half as efficient as *aa*_3_ (Von Ballmoos et al., 2012). The expression could be due to the culturing conditions for this trial. A simple shake flask batch was used, which could have led to some oxygen limitation at the end of the batch phase, forcing *C. thermarum* TA2.A1 to adopt a more austere lifestyle. Such questions could be solved by culturing under more controlled conditions, such as a chemostat. We would expect that culturing at lower dissolved oxygen would also trigger the expression of the other two terminal oxidases, namely cytochromes *bb*_3_ and *bd*.

In terms of carbon import machinery, a range of different transporters was detected, pertaining to the SugABC, RbsABC, MalEFGK, DctPQM, and UgpABC complexes (Figures 2A, 3). Considering the use of sucrose as a carbon source, the detection of multiple subunits of SugABC was expected (Figure 2B). MalEFGK is annotated as a maltose importer but might transport sucrose as well; this dual specificity is described in *Streptococcus mutans* (Kilic et al., 2007). The detection of possibly two sucrose importers means this research has failed to highlight the genes encoding for the sucrose:sodium symporter that originally sparked the interest in *C. thermarum* TA2.A1 (Peddie et al., 1999, 2000). The detection of the TRAP-type C4-dicarboxylate importers DctPQM, RbsABC complex, and UgpABC complex (Figures 2A, 3) may be due to their scavenging functions rather than their favored presence under our culturing conditions. In line with other alkaliphiles, no defined medium for *C. thermarum* TA2.A1 exists, and it still requires the presence of tryptic peptone extract to grow, which could contain a substrate for all of these transporters. In agreement with this proposition, several amino acid importers, oligopeptide importers, and importers of related compounds, like glutathione, were also detected. Finally, the proteins translocase SecDF and the flagellum proteins were also detected.

**Figure 4:**
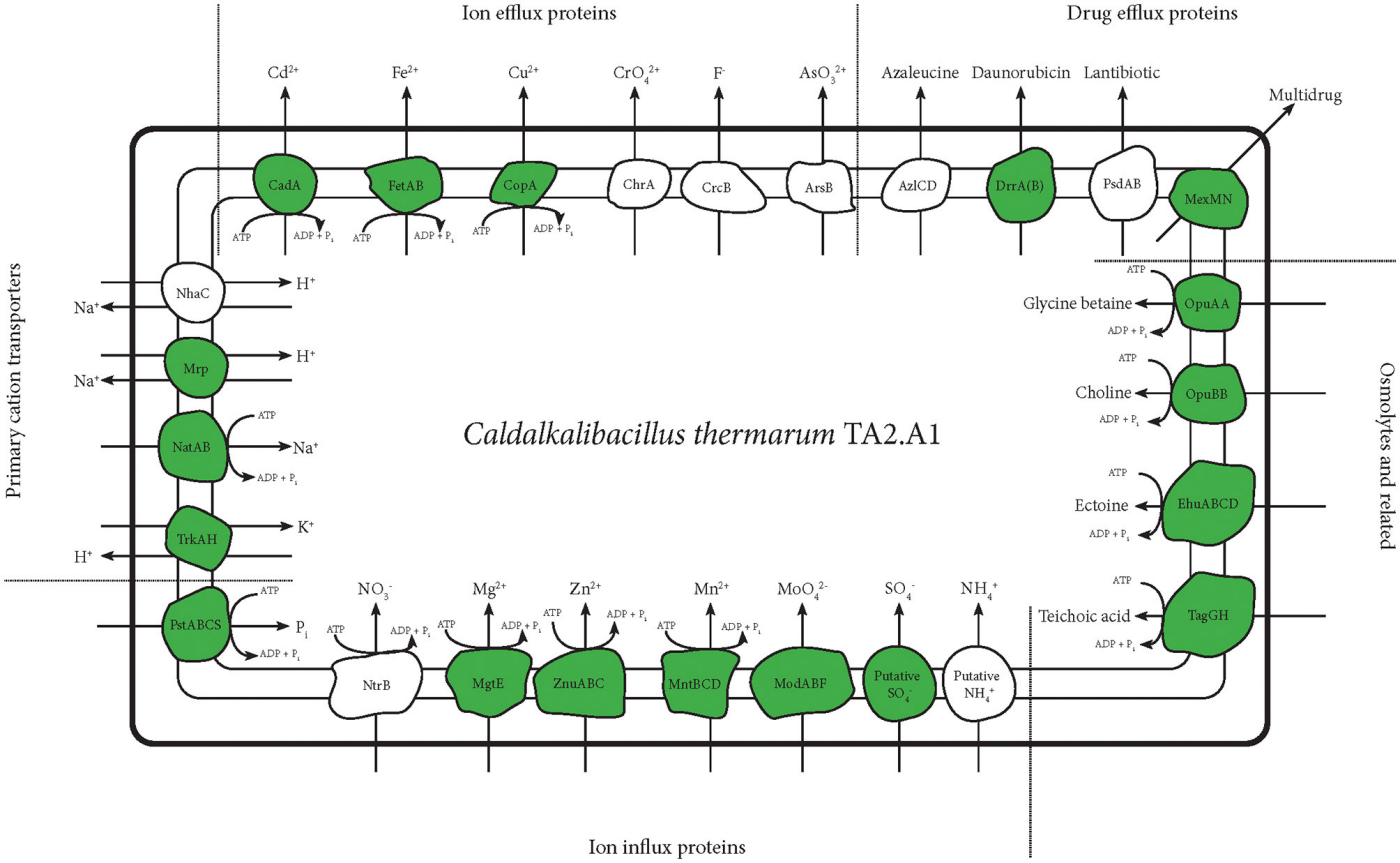
An overview of transporters detected with any of the abovementioned protocols. This figure selectively shows transporters of metals and inorganic cations and anions. Transporters related to drug efflux and osmolyte import are also shown. The teichoic acid importer is displayed, being regarded as an important protein for alkaliphiles (Aono and Uramoto, 1986; Aono et al., 1995, 1999), even though previous tests showed teichoic acid presence in *C. thermarum* TA2.A1 (Kleikamp et al., 2020). Detected proteins are depicted in green.

Homeostasis of monovalent cations is crucial for any alkaliphile, and generally, this is coupled with proton transport in an antiport protein (Krulwich and Cheng, 1994). *C. thermarum* TA2.A1 has two types of sodium:proton antiporters; a few subunits of the multisubunit Mrp were detected under the conditions used. The active ATP powered NatABC was also expressed. *C. thermarum* TA2.A1 relies on the TrkAH system for potassium uptake. The low-affinity TrkAH system found in most bacteria has been highlighted as a strategy to combat saline stress (Kraegeloh et al., 2005; Guo et al., 2009). *C. thermarum* TA2.A1 is capable of growing up to 6% NaCl (Xue et al., 2006), perhaps in part due to its reliance on this potassium uptake system. Two proteins pertaining to the A subunit of the potassium:proton antiporter TrkAH were detected (Figures 2B, 4); the *trkA* genes encoding for the expression of these proteins are not part of a single operon or are they close to any of the other *trkA* or *trkH* genes. As an importer of inorganic phosphate, the subunit S of the Pts complex was detected only in the experiments using alternative digestion enzymes (Figures 2B, 4). For the import of trace metals, transporters for zinc, molybdate, magnesium, and manganese were detected (Figure 4).

Other metal transporters, such as cadmium, copper and iron, were also detected. Notably, these were all described as exporters (Yoon and Silver, 1991; Rensing et al., 2000). The ferrous exporter FetAB was previously described as an exporter and implied as an important protein in avoiding problems with radical formation (Nicolaou et al., 2013). Several drug exporters were also detected, further shedding light on the possible mechanisms *C. thermarum* TA2.A1 might use to remain dominant in a given environment. Finally, several importers related to osmolytes were detected. The incorporation of transporters of glycine betaine and ectoine could be another method used by *C. thermarum* TA2.A1 to combat the challenges posed by the high pH at which it lives. Especially glycine betaine was abundantly available in the rich medium used; the strategy of utilizing compatible solutes should be perfectly viable.

This study is the first report on the proteome of *C. thermarum* TA2.A1, in which 1,398 proteins (45.3% of all potential proteins) were detected in at least one of the experiments. Of this, 158 contain at least a single TMH (11.3% of all identified proteins) and are therefore considered membrane proteins. The total coverage of the membrane proteome is 20.9%. *C. thermarum* TA2.A1 is an alkaliphile, and therefore this study focuses primarily on proteins identified in the ETC and on transporters. For an alkaliphile, homeostasis of protons, sodium, and potassium is crucial, and this study detected transporters for all of these compounds. Importers of various compounds related to central carbon metabolism and anabolism were also detected, as well as a complete ETC. Combined, this study gives a comprehensive overview of the membrane proteins required by an alkaliphile to survive and provides a valuable benchmark for further research on this organism. The authors specifically note its adaptability to a broad pH range as an area for further research. The conditions under which *C. thermarum* TA2.A1 employs its alternative terminal oxidases are also yet to be revealed. In that regard, performing solubilisation of membrane extracts as an addition to whole cell lysate proteomics will increase the chances of detecting these alternative terminal oxidases.

## Conclusion

Here we report on a proteomic study where we investigated several sample preparation approaches to maximize the coverage for membrane proteins for *C. thermarum* TA2.A1. The whole cell proteomics experiment combined with proteomics of solubilised membrane extracts yielded 1,398 proteins (45.3% of the total proteome), of which 158 (11.3% of the discovered proteins; 20.9% of the membrane proteome) were membrane proteins. Interestingly, all different membrane solubilisation methods provided a unique set of membrane proteins. In terms of solubilisation of membrane proteins, we conclude that treatment with SDS at 50°C and the analysis of a sample treated with FOS-choline-12 at 65°C or 80°C proved most effective. The modest effect of alternative protease digestion, despite the promising *in silico* analysis, is a subtle but crucial warning that alternative complications, such as shielding of potential digestion sites, contain post-translational modifications such as glycosylations and lipidations.

The entire optimal ETC was detected using the combined methods for analysis, as well as two less efficient proteins – the Ndh-2 as an “alternative Complex I” (Biagini et al., 2006; Elguindy and Nakamaru-Ogiso, 2015) and the cytochrome oxidase *ba*_3_ as an alternative Complex IV. Additionally, we detected all proteins required for the transport of protons, sodium, and potassium, whose homeostasis is crucial to an alkaliphile. Finally, the expression of the glycine betaine and ectoine importers implies that *C. thermarum* TA2.A1 relies in part on osmolytes for its survival, something that has not been reported previously in the literature for this organism. Overall, this research has given a comprehensive overview of proteins that we expect to be expressed and shed light on some additional potentially interesting proteins. In short, this research provides a valuable benchmark for further research into alkaliphiles and *C. thermarum* TA2.A1 in particular.

## Data availability statement

Mass spectrometric raw data have been deposited to the ProteomeXchange Consortium via the PRIDE partner repository and are publicly available under the project code PXD042369.

## Author contributions

SJ, DS, ML, MP, and DM conceptualized the study and wrote the manuscript. SJ performed biological work and extractions. MP performed proteomics experiments. All authors contributed to the article and approved the submitted version.

## Supplementary material

Supplementary Figure 1 SDS-PAGE gel (Any kD^TM^ Mini_PROTEAN® TGX^TM^, Bio-Rad) of extracted membranes. Ladder (Precision Plus Protein^TM^ Unstained Protein Standards, Bio-Rad) was loaded on either side of the samples; 1× pass indicates a sample of extracted membranes that only passed through the cell disruptor once, while 2× pass indicates a sample of extracted membranes that passed through the cell disruptor twice, confirming the regular protocol. Samples were diluted beforehand in MilliQ water to ensure 5 μg protein was loaded in the well. Samples were dyed with a mix of 2-mercaptoethanol and 4× Leammli Sample Buffer (Bio-Rad) according to the manufacturer's specifications. After running at 200 V for 45 min, the gel was submerged in SimplyBlue^TM^ Safe Stain (Invitrogen) for 2 h and subsequently destained overnight in a 5% methanol and 7.5% acetic acid solution. This picture was taken afterwards.

Supplementary Figure 2 Violin plot showing an *in silico* digestion profile of the *C. thermarum* TA2.A1 membrane proteome using different digestion enzymes: Trypsin, LysC, chymotrypsin, and combining LysC with chymotrypsin. Sizes are relative to the number of peptides, distributed by peptide length over the *y*-axis. Horizontal lines indicate the range of peptide lengths (from 7 to 35 amino acids) considered ideal for detection by LC-MS.

Supplementary Table 1 Raw mass spectrometry data of all performed conditions. The absolute peak area and the number of unique peptides are shown for all proteins detected per condition. Some column headers are self-explanatory, as the name directly refers to a specific condition. For those that do not, the order is as follows: FOS, solubilised at 65°C (S1); FOS, solubilised at 65°C (S2); FOS, solubilised at 50°C (S3); FOS, solubilised at 65°C (S4); FOS, solubilised at 80°C (S5); SDS, solubilised at 50°C (S6); SDS, solubilised at 65°C (S7); and SDS, solubilised at 80°C (S8).

Supplementary Table 2 The locus tag, protein id, gene, and subunit identifier (Accession: CP082237) for proteins are shown in Figures 2–4. An arbitrary decision was made as to which complexes, aside from the ETC, should be included in Figures 2A, B, 3, 4 respectively.
