# Supplementary figures and images for: Membrane proteome of the thermoalkaliphile Caldalkalibacillus thermarum TA2.A1
Source: Front Microbiol. 2023 Jul 28;14:1228266. doi: 10.3389/fmicb.2023.1228266 (PMC10416648; doi:10.3389/fmicb.2023.1228266)

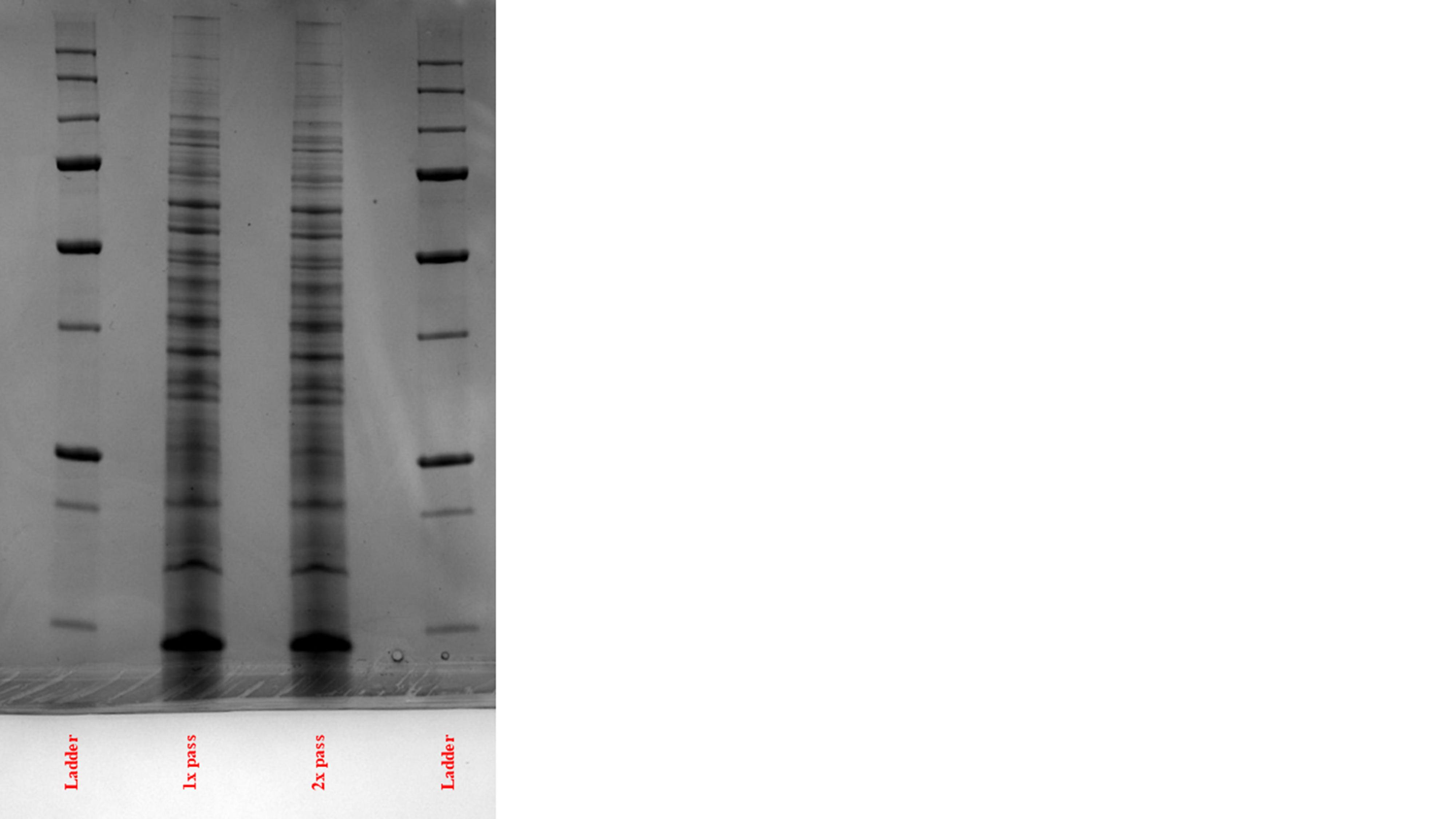

Supplement: Supplementary Figure 1 — SDS-PAGE gel (Any kDTM Mini_PROTEAN® TGXTM, Bio-Rad) of extracted membranes. Ladder (Precision Plus ProteinTM Unstained Protein Standards, Bio-Rad) was loaded on either side of the samples; 1× pass indicates a sample of extracted membranes that only passed through the cell disruptor once, while 2× pass indicates a sample of extracted membranes that passed through the cell disruptor twice, confirming the regular protocol. Samples were diluted beforehand in MilliQ water to ensure 5 μg protein was loaded in the well. Samples were dyed with a mix of 2-mercaptoethanol and 4× Leammli Sample Buffer (Bio-Rad) according to the manufacturer's specifications. After running at 200 V for 45 min, the gel was submerged in SimplyBlueTM Safe Stain (Invitrogen) for 2 h and subsequently destained overnight in a 5% methanol and 7.5% acetic acid solution. This picture was taken afterwards. [file Image_1.TIF]

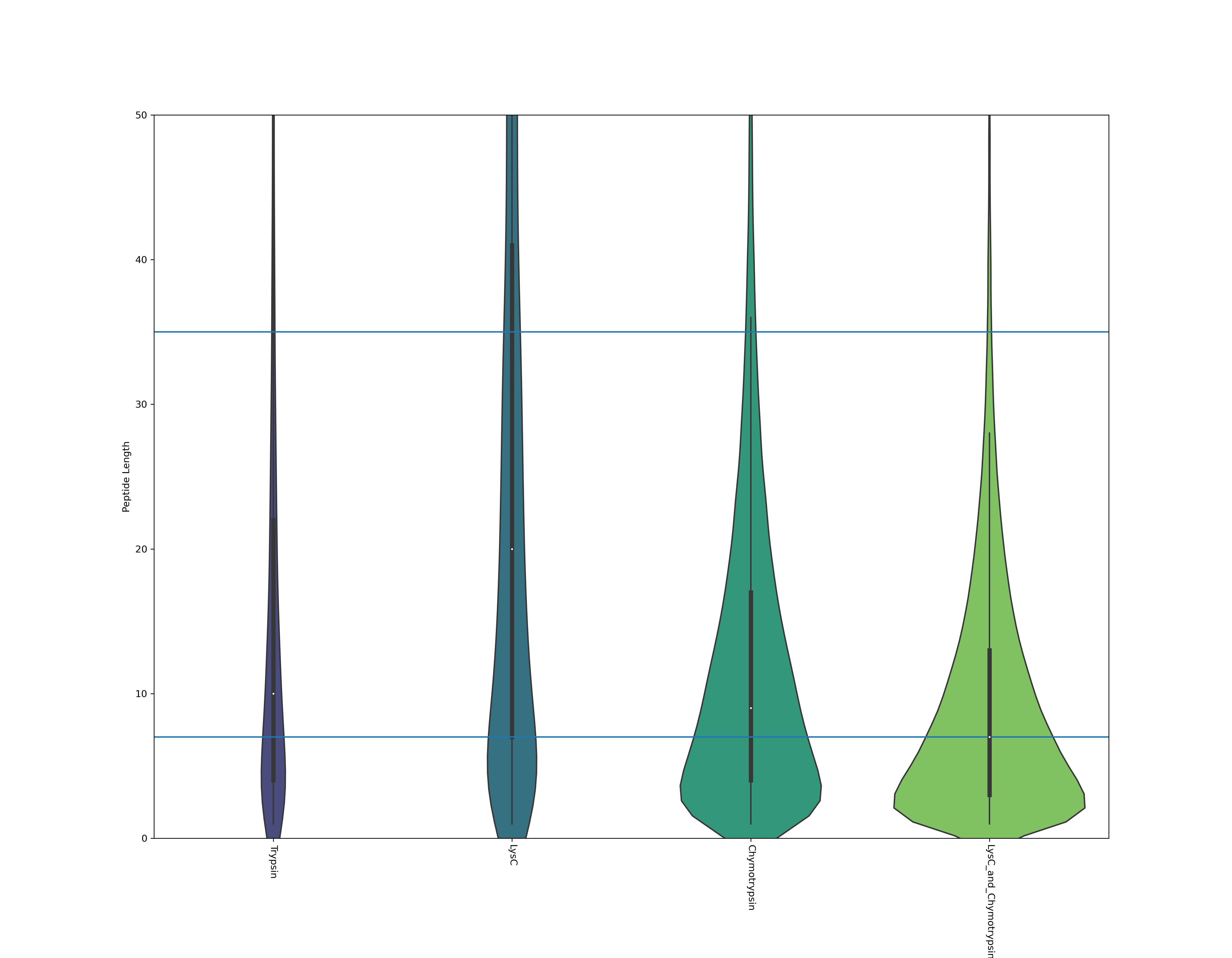

Supplement: Supplementary Figure 2 — Violin plot showing an in silico digestion profile of the C. thermarum TA2.A1 membrane proteome using different digestion enzymes: Trypsin, LysC, chymotrypsin, and combining LysC with chymotrypsin. Sizes are relative to the number of peptides, distributed by peptide length over the y-axis. Horizontal lines indicate the range of peptide lengths (from 7 to 35 amino acids) considered ideal for detection by LC-MS. [file Image_2.JPEG]
